# Exploring eye care pathways, patient priorities and economics in Pakistan: A scoping review and expert consultation study with thematic analysis

**DOI:** 10.1111/opo.12977

**Published:** 2022-03-23

**Authors:** Manal Malik, Niall Strang, Pauline Campbell, Sven Jonuscheit

**Affiliations:** ^1^ Department of Vision Sciences Glasgow Caledonian University Glasgow UK; ^2^ Nursing, Midwifery and Allied Health Professions Research Unit Glasgow Caledonian University Glasgow UK

**Keywords:** blindness, eye care services, Pakistan, scoping review, thematic analysis, visual impairment

## Abstract

**Purpose:**

As the prevalence of eye diseases increases, demand for effective, accessible and equitable eye care grows worldwide. This is especially true in lower and middle‐income countries, which have variable levels of infrastructure and economic resources to meet this increased demand. In the present study we aimed to review the literature on eye care in Pakistan comprehensively, with a particular focus on eye care pathways, patient priorities and economics.

**Methods:**

A systematic scoping review was performed to identify literature relating to eye care in Pakistan. Searches of relevant electronic databases and grey literature were carried out. The results were analysed through a mixed methods approach encompassing descriptive numerical summary and thematic analysis. To consolidate results and define priority areas for future study, expert consultation exercises with key stakeholders were conducted using qualitative semi‐structured interviews.

**Results:**

One hundred and thirty‐two papers (published and unpublished) were included in the final review. The majority (*n* = 93) of studies utilised a quantitative design. Seven interlinked themes were identified: eye care pathways, burden of eye disease, public views on eye‐related issues, workforce, barriers to uptake of eye care services, quality of eye care services and economic impact of blindness. Research priorities included investigating the eye care workforce, the quality and efficiency of current eye care services, eye care services available in rural Pakistan and the costs and benefits related to eye care provision and sustaining eye care programmes.

**Conclusions:**

To the best of our knowledge, this is the first review to synthesise evidence from papers across the field relating to eye care in Pakistan. As such, this work provides new insights into the achievements of the national eye health programme, challenges in eye care in Pakistan and priority areas for future research.

**Key points**

- There is currently a lack of high‐quality and qualitative research regarding pathways, patient priorities and the economic aspects of eye care in Pakistan.
- Analysis of the research surrounding eye care in Pakistan generated seven inter‐related themes which formed the basis of a framework that may be developed further into an evaluation tool to assess local eye care services.
- The review highlights achievements to date surrounding eye care in Pakistan, and identifies research priorities including investigating the eye care workforce, the quality and efficiency of current eye care services, eye care services available in rural Pakistan and the costs and benefits related to eye care provision and sustaining eye care programmes.

## INTRODUCTION

Effective and accessible eye care can make a significant difference to the lives of millions of people as the majority of all causes of visual impairment are preventable or curable.^1^ Eye care has been identified by the World Health Organisation (WHO) as a global health priority. In 2013, the WHO published an ambitious action plan to reduce the prevalence of avoidable visual impairment (VI) by 25% by 2019.^1^ Despite many successful initiatives, this target has not been achieved, and recent studies have shown that the prevalence of blindness and moderate and severe VI worldwide continues to increase, and is now estimated at 4.34%.^2^

The WHO world report on vision^3^ outlines the challenges present in terms of eye care worldwide. Examples of such challenges include inequities in the availability and quality of prevention, treatment and rehabilitation services; a lack of trained eye care professionals; greater demand on existing eye care services due to the increasing prevalence of VI and poor integration of eye care services into health systems. These challenges are heightened in low and middle‐income countries, as evidenced by a four‐fold higher prevalence of avoidable VI when compared to high‐income countries. The report provides recommendations for action to improve global eye care services, with the main proposal being that all countries work towards providing integrated people‐centred eye care services, ensuring that patients receive a continuum of eye care based on their individual needs throughout their lives. However, achieving integrated people‐centred eye care services is a greater challenge for low and middle‐income countries, which already lack the infrastructure and resources to deal with the current demand placed on eye care provision.^3^, ^4^

Recent studies estimate that more than 21 million people in Pakistan still suffer from blindness and visual impairment, with a prevalence of blindness (presenting visual acuity worse than 3/60 in the better eye) of 0.9%.^8^, ^14^ which is considerably higher than that found in high‐income countries as well as eastern and central Europe (0.15%).^7^ Cataract accounts for over half of the cases of avoidable blindness in Pakistan, but corneal opacity (11.8%), uncorrected aphakia (8.6%) and glaucoma (7.1%) are also significant contributors.^8^, ^9^ However, these prevalence numbers were last published in 2006,^8^ and Rapid Assessments of Avoidable Blindness (RAAB) are currently being conducted in Pakistan, with outcomes likely to become available in 2022. At this point in time, only assumptions can be made as to whether rates have risen or declined. In neighbouring India, data from a recent large scale RAAB has been released showing an estimated overall reduction in the prevalence of blindness of 1.99%.^10^ Nonetheless, due to the aging population in India, the prevalence of age‐related eye conditions including glaucoma and macular degeneration has increased, while cataract accounts for 66.2% of avoidable blindness.^10^, ^11^ Pakistan's population is similarly aging, with 6.8% over 60 years of age, a figure that is expected to climb to 12.3% by 2051, suggesting that comparable trends in the prevalence of age‐related eye disorders may be noticed.^5^ While major advancements towards providing comprehensive, integrated eye care services in Pakistan are underway – by upgrading facilities, workforce training and raising awareness^12^ – eye care provision remains a significant health care challenge.^8^, ^13^

Despite the implementation of a number of successful initiatives to reduce avoidable blindness, namely district comprehensive eye care services,^12^ it is not clear what additional steps should be taken to improve the eye care system in Pakistan further, and research in the field is hampered by a lack of synthesised evidence about the efficacy, equity and accessibility of eye care. Previous reviews in this area focused on synthesising prevalence studies in Pakistan covering vision loss,^24^ refractive error,^25^ diabetic retinopathy (DR),^26^ coverage of cataract surgical services^27^ and keratoconus.^28^ Therefore, a review with a broader and comprehensive remit is required to provide an overview of the entire field surrounding eye care services in Pakistan, highlighting the most pressing needs and priority areas for future research.

Moreover, the United Nations (UN) have outlined 17 sustainable development goals (SDGs) as a global call for action to create a better future for all people and the environment by 2030.^29^ Vision contributes significantly to the 2030 Agenda for Sustainable Development, and intersects with a number of SDGs including, poverty reduction (SDG 1), supporting well‐being and ensuring healthier lives for everyone at all ages (SDG 3), education (SDG 4), economic growth and employment (SDG 8), gender equality (SDG 5) and reducing inequality within and amongst countries (SDG 10). The following scoping review and subsequent expert consultation contribute to these SDGs by providing an overview of eye health in Pakistan and suggestions to achieve the goals set out by the UN.

In this study, we aim to review the research on eye care services in Pakistan comprehensively, to identify, appraise and synthesise the current research, and to provide the evidence‐base that will allow the identification of priority areas for further research as well as opportunities to improve eye care. Specifically, we sought to answer the research question ‘What is known from the existing literature about current pathways, patient priorities and economics in terms of eye care in Pakistan?’

## METHODS

### Study design

We conducted a systematic scoping review following the six‐stage methodological framework.^16^, ^17^, ^18^ This included: (i) identifying the research question, (ii) identifying the relevant literature, (iii) selection of studies, (iv) charting the data, (v) collating, summarising and reporting the data using current reporting guidance and (vi) consultation. A protocol detailing each of the steps was developed a priori and is available from the authors on request. The specific methods for each of the six steps are briefly outlined below.

#### Identifying the research question

A scoping review was selected as a review method as it allows for literature surrounding eye care in Pakistan to be ‘mapped.’ The process of ‘mapping’ allows one to summarise evidence to expose the extent, nature and range of knowledge within a field.^16^, ^19^ According to Arksey and O'Malley,^16^ a wide approach should be taken when selecting overarching research questions in order to generate breadth of coverage. Therefore, a broad question was formulated with mention of care pathways, patient priorities and economics relating to eye care in Pakistan.

#### Identifying the relevant literature: search strategy

A comprehensive search strategy was initially developed by the principal author (MM) in Medline (EBSCOhost) using a combination of keywords and MeSH headings for: (a) eye care pathways, (b) patient priorities, (c) economics, (d) workforce and (e) Pakistan. Each of the five topics were combined in different varieties to produce the relevant results. The search was developed and checked by an information specialist (KM) in accordance with PRESS guidelines.^20^ This was adapted for the other electronic databases. The search string is shown in File 1.

##### Electronic searches

We systematically searched the following major electronic databases:

- Medical Literature Analysis and Retrieval System Online (Medline, EBSCOhost) (date of last search: 9 June 2020);
- The Cumulative Index to Nursing and Allied Health Literature (CINAHL) (date of last search: 10 June 2020)
- Web of Science (date of last search: 10 June 2020).

##### Supplementary searches

In addition to searching electronic databases, we conducted supplementary searches of Non‐Governmental Organisation websites including:

- Sightsavers (sightsavers.org)
- Fred Hollows Foundation (hollows.org)
- World Health Organisation (WHO) (who.int)
- Google and Google Scholar (scholar.google.com)
- Pakistan Department of Health website (nhsrc.gov.pk)
- Pakistani Government online archives (archives.gov.pk)
- Key journals (Community Eye Health, Ophthalmic Epidemiology, Ophthalmology Pakistan and Journal of the College of Physicians and Surgeons Pakistan)

We also conducted backward citation tracking of all included studies and engaged with key stakeholders (see section vi) to identify any potentially relevant studies.

#### Study selection

Study eligibility criteria is summarised in Table 1.

**TABLE 1:** Eligibility criteria

	Inclusion criteria	Exclusion criteria
Population	All ages.	None
Interest	Relevant to eye care. Epidemiology studies on eye related conditions.	No relevance with any aspect of eye care.
Context	Pakistan or international study with specific mention or figures for Pakistan.	No specific mention of Pakistan.
Study design	All study designs.	None
Other	English only from 1994 onwards. Published and unpublished (grey) literature.	Not published in English. Published before 1994.

One explicit inclusion criterion is that the studies selected are all in English and not in other languages such as Urdu. This is prudent as it helps to overcome linguistic limitations of the authors and control time and cost constraints associated with translation of non‐English studies. Since the accepted academic language at undergraduate and higher levels in Pakistan is English, applying this language limitation is unlikely to have a major impact on the results.^157^

**FIGURE 1:**
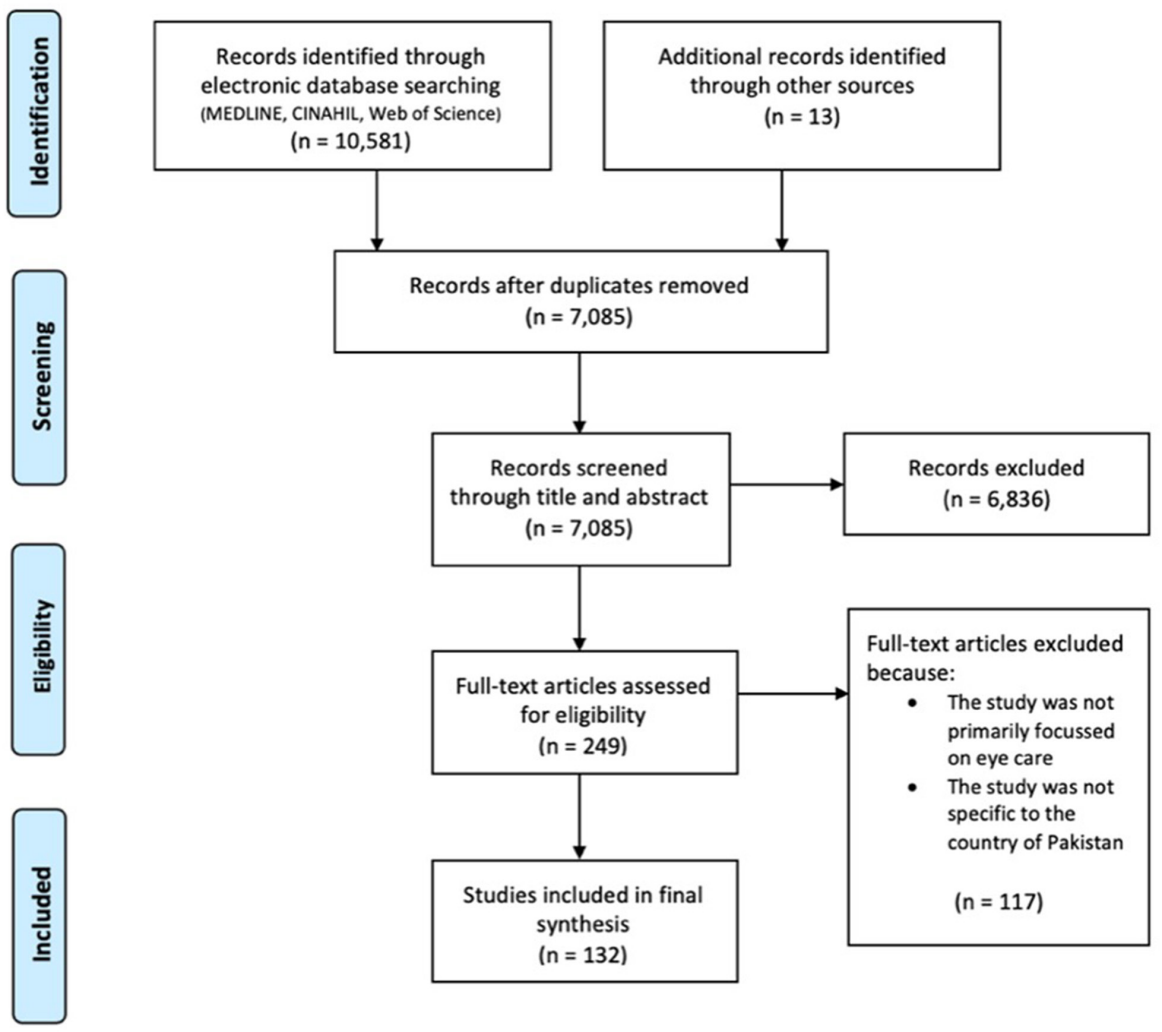
Adapted preferred reporting items for systematic reviews and meta‐analyses (PRISMA) study flow diagram.^21^ Detailed flow of information through different phases of study selection maps the number of papers identified, including reasons for exclusion.

Records were imported into reference manager software (EndNoteX9.3.3, Clarivate, clarivate.com) and de‐duplicated. Titles and abstracts were screened by one reviewer (MM), using the selection criteria (Table 1); the remaining studies were imported into Covidence for full text screening. A series of training exercises were initially undertaken to ensure reliability between reviewers. Three reviewers (MM, SJ, NS [with SJ & NS working as a pair]) independently screened full texts. Conflicts were resolved initially by discussion or involving a third reviewer. The flow of the studies is shown in Figure 1.^21^

#### Charting the data and coding

##### Data extraction

A draft charting form was piloted by three reviewers independently using a sample of five articles. The following data was extracted for each study:

- Study characteristics (i.e., author, date of publication)
- Aims of the study
- Methods including study design, methods used to conduct the study, duration of study, sample size
- Geographical region (i.e., province)
- Age group of participants
- Details about the workforce (i.e., profession, training) and sector (e.g., public, private)
- Key findings

Data were extracted by one reviewer (MM), cross checked by a second reviewer (SJ, NS) and any disagreements were resolved by discussion, with assistance from a third reviewer if necessary.

##### Coding key themes from included studies

Due to the broad, inclusive approach adopted, the papers were initially grouped according to their topic subject. A coding exercise was carried out based on a framework outlined by Braun and Clarke.^22^ One paper from each subject was selected at random to undergo coding; line by line coding was then carried out manually. Eleven papers were coded to produce an initial thematic framework. The remaining studies (*n* = 121) were then assigned a primary and, where needed, a secondary theme. Secondary themes were found to be required for articles that covered more than one key topic or that were relatively broadly focused. Sub‐themes were also assigned where appropriate to help organise papers within themes. As coding of the remaining papers progressed, new themes that were identified were incorporated into the framework, and previously coded studies were re‐assessed and updated.

#### Collating, summarising and reporting the results

Descriptive data are displayed using bar and radar charts, histograms, maps and tables. Key findings from the included studies were brought together within a narrative synthesis. Data relating to primary themes were brought together alongside key stakeholder opinions (see Consultation).

#### Consultation

We conducted an expert consultation exercise with clinical optometrists, some of whom were also academic researchers (referred to as ‘stakeholders’). Four participants identified through professional networks were selected based on their clinical experience working within the field of eye care in Pakistan. National and provincial co‐ordinators for eye health from the Ministry of Health in Pakistan were also invited to provide comments. Following consent from participants, a series of recorded semi‐structured interviews were undertaken one‐to‐one via an online communication platform (Zoom Video Communications, zoom.us) Ethical approval was granted on 7 December 2020 by the Glasgow Caledonian University School of Health and Life Sciences Research Ethics Committee (HLS/LS/A20/030). During the consultation exercise we asked the stakeholders whether the findings from the literature were consistent with their real‐world perspective, and sought information about stakeholders' own clinical and research priorities based on their context of work. Stakeholders were also asked if they were aware of any additional literature relevant to eye care in Pakistan that was not picked up by our searches.

##### Quality assessment

The review included a variety of studies which had used a range of research methods; therefore, the “QualSyst” tool was chosen to evaluate the quality of the included studies.^23^ The tool consists of two scoring checklists (qualitative and quantitative). A final numerical summary score can be calculated which gives a value from 0.00 (lowest quality score) to 1.00 (highest quality score).

For the present scoping review, three reviewers carried out a trial quality assessment using the checklist and generated a summary score independently based on a random selection of 13 articles. Initial agreement was reached in around half of the papers and considered too low for the main analysis. Evaluating reasons for the low agreement, it became evident that one reviewer was marking considerably more strictly than the other two. This was discussed, and a collective training session undertaken to ensure consistency amongst all reviewers. For mixed method approaches, both checklists were applied to the paper, and the final result was the average of the two summary scores. Below are the defined thresholds which were applied in this review to ensure consistency:

- >0.80 = Strong
- 0.71–0.79 = Good
- 0.50–0.70 = Adequate
- <0.50 = Poor

## RESULTS

### Study selection

Our searching identified 10,594 records; 3509 were excluded as they were duplicate records. From the remaining 7085 records, 6836 were excluded as they were irrelevant records. Two independent reviewers screened the remaining 249 full text papers, of which 117 documents were excluded as they were either not based in Pakistan or focused on eye care, leaving a total of 132 documents that met the selection criteria. Out of the 132 documents 112 were regarded as research studies with the remaining 20 documents encompassing news items and unpublished reports. In the following sections we refer to the 112 documents as ‘studies’ and the 20 documents without a clear research method as ‘other relevant documents,’ and where both are combined, we refer to the two combined categories as ‘papers.’

### Description of included papers

Key details of the 132 included papers are summarised in Table 2.

**TABLE 2:** Key details of 132 papers, organised by theme

Paper characteristics		All papers N = 132	Eye care pathways, N = 47	Burden of eye disease, N = 20	Public views on eye related issues, N = 19	Workforce, N = 15	Barriers to uptake of eye care services, N = 15	Quality of eye care services, N = 13	Economic impact of blindness, N = 3
Research method	Quantitative (*n*, %)	93 (71)	34 (37)	20 (22)	12 (13)	5 (5)	10 (11)	10 (11)	2 (2)
	Qualitative (*n*, %)	8 (6)	3 (38)	0	1 (12)	2 (25)	2 (25)	0	0
	Mixed methods (*n*, %)	12 (9)	2 (17)	0	6 (50)	4 (33)	0	0	0
	N/A (*n*, %)*	19 (14)	8 (42)	0	0	4 (21)	3 (16)	3 (16)	1 (5)
Design	Cross‐sectional (*n*, %)	70 (53)	29 (41)	9 (13)	13 (19)	7 (10)	6 (9)	5 (7)	1 (1)
	Cross‐sectional cohort (*n*, %)	10 (8)	2 (20)	2 (20)	2 (20)	1 (10)	2 (20)	1 (10)	0
	Cohort (*n*, %)	6 (5)	0	3 (50)	1 (17)	0	1 (17)	1 (17)	0
	Case report (*n*, %)	9 (7)	5 (56)	0	0	2 (22)	0	0	2 (22)
	Case series (*n*, %)	7 (5)	2 (29)	1 (14)	1 (14)	0	1 (14)	2 (29)	0
	Case control (*n*, %)	1 (1)	0	0	1 (100)	0	0	0	0
	Systematic review (*n*, %)	3 (2)	0	2 (67)	0	0	1 (33)	0	0
	Meta‐analysis (*n*, %)	1 (1)	0	0	0	0	1 (100)	0	0
	Literature review (*n*, %)	1 (1)	0	1 (100)	0	0	0	0	0
	News item (*n*, %)	12 (9)	3 (25)	0	0	3 (25)	3 (25)	3 (25)	0
	Other** (*n*, %)	12 (9)	6 (50)	2 (17)	1 (8)	2 (17)	0	1 (8)	0
Epidemiology study	Yes (*n*, %)	55 (42)	26 (47)	17 (31)	2 (4)	1 (2)	6 (11)	2 (4)	1 (2)
	No (*n*, %)	77 (58)	21 (27)	3 (4)	17 (22)	14 (18)	9 (12)	11 (14)	2 (3)
Setting	Public (*n*, %)	37 (28)	17 (46)	4 (11)	5 (14)	4 (11)	2 (5)	4 (11)	1 (3)
	Private*** (*n*, %)	24 (18)	9 (38)	4 (17)	3 (13)	2 (8)	2 (8)	3 (13)	1 (4)
	Both (*n*, %)	19 (14)	6 (32)	0	4 (21)	5 (26)	3 (16)	1 (5)	0
	Not Recorded (*n*, %)	52 (39)	15 (29)	12 (23)	7 (13)	4 (8)	8 (15)	5 (10)	1 (2)

* ‘Other relevant documents’ were included in this category which comprised mainly of news items and unpublished reports which did not have a defined research method.

** The category ‘other’ consisted of observational studies, statistical prediction, text and opinion pieces, project plans, mid‐term reviews, learning reviews and an end line project evaluation.

*** Private setting included private sector service providers and non‐governmental organisations (NGOs). These two were combined rather than having an additional category for the voluntary sector, as in Pakistan a number of private hospitals provide both private care and not‐for profit services depending on an individual's income and financial status. Additionally, a number of papers carried out in these hospitals did not specify whether they were treating paying or non‐paying patients, making it difficult to distinguish between the private and voluntary categories.

#### Geographical distribution

**FIGURE 2:**
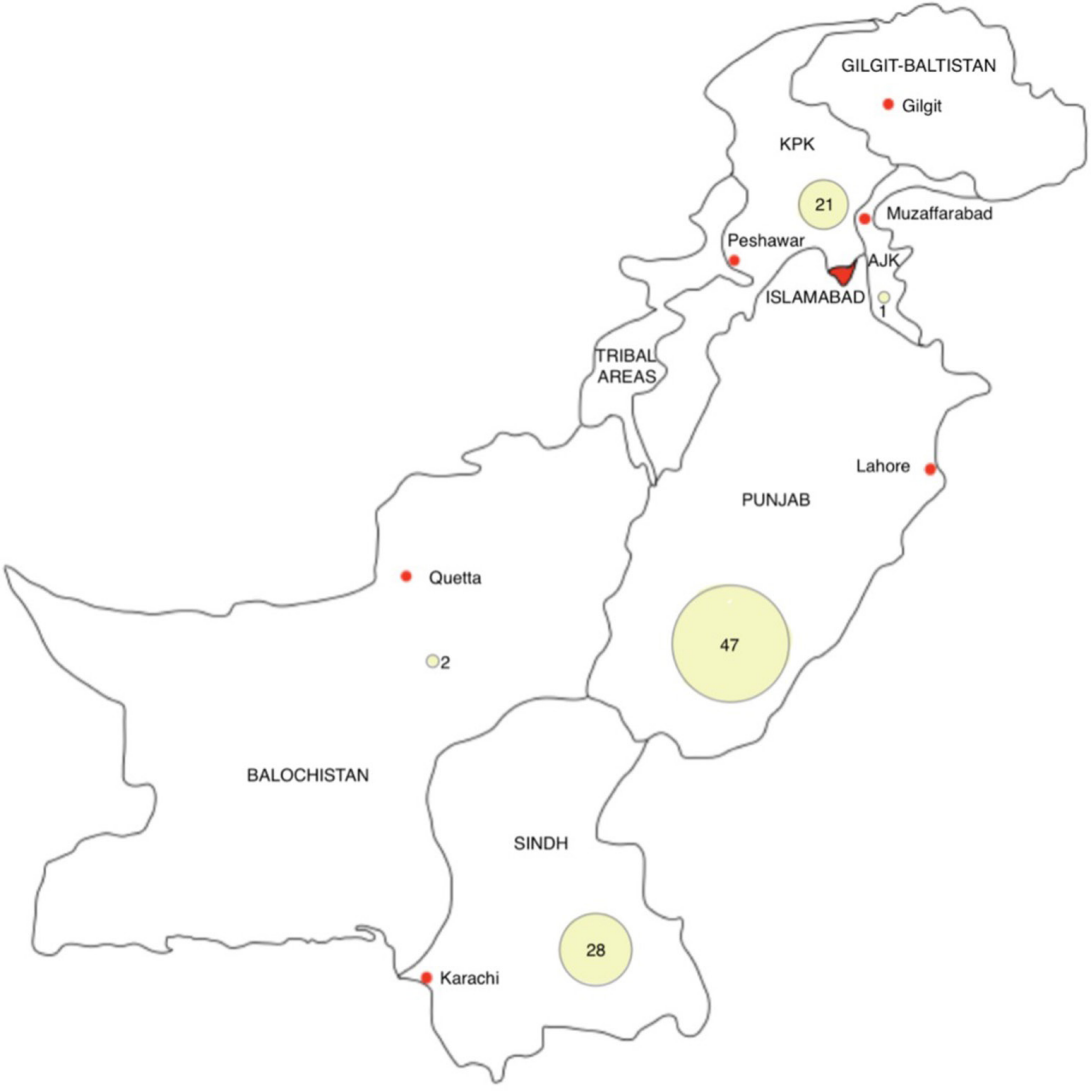
Map of Pakistan. Yellow bubbles represent distribution of the papers and are accompanied by the number of papers carried out in each province. KPK = Khyber Pakhtunkhwa, AJK = Azad Jammu and Kashmir

The geographical distribution of the papers is shown in a map produced by the lead author in Figure 2. Thirty‐eight of the papers were carried out nationwide, seven were performed in more than one province and for three the provincial location was not mentioned. For papers reporting on regional aspects of eye care (i.e., in provinces), the majority (*n* = 47) were undertaken in the Punjab province, followed by Sindh (*n* = 28), Khyber Pakhtunkhwa (KPK) (*n* = 21), Balochistan (*n* = 2) and Azad Jammu and Kashmir (*n* = 1), respectively. There was no specific mention of any paper solely being undertaken in the province of Gilgit‐Baltistan, Islamabad or in the Tribal Areas.

#### Year of publication

**FIGURE 3:**
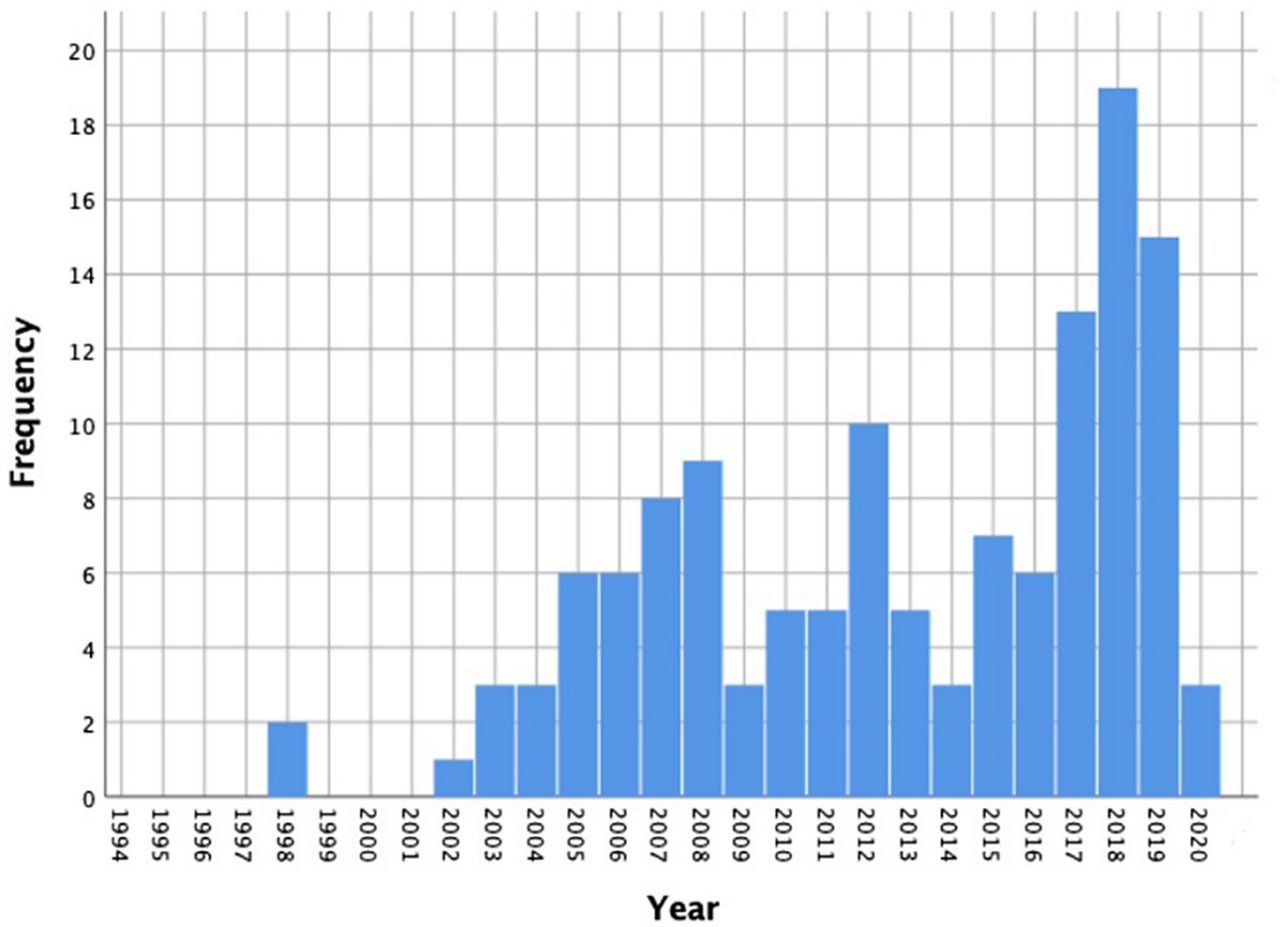
Histogram showing distribution of papers published from 1994 to 2020

The year of publication of the included papers ranged from 1998 through to 2020 (Figure 3). The number of papers published has not been consistent over this period, with a drop in publication in 2009 and again in 2014. The year in which the most papers were published was 2018.

#### Quality appraisal of included studies

**FIGURE 4:**
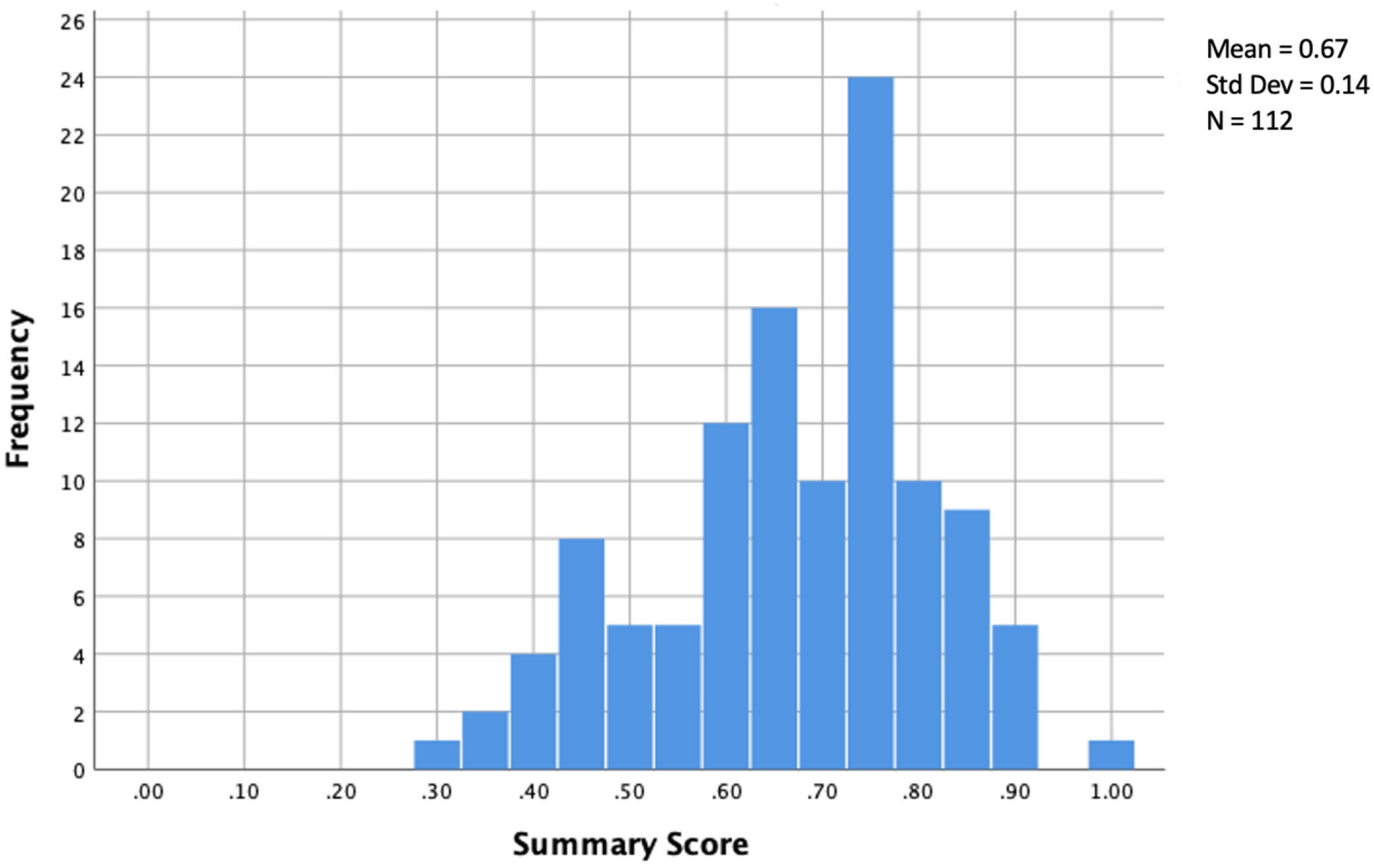
Histogram showing distribution of frequency of quality summary scores using the QualSyst tool on 112 studies

A total of 112 studies were eligible for quality appraisal. The mean (±SD) quality appraisal score was 0.67 ± 0.14 and the most frequent summary score (mode) was 0.73, with 11 papers sharing this value. The range extended from 0.30 to 1.00 (Figure 4). The Shapiro–Wilk test of normality showed a *p*‐value of <0.05 showing that the quality scores were not normally distributed. Studies were then assigned a quality rating; the most common quality rating was adequate (*n* = 45), followed by good (*n* = 28), strong (*n* = 24) and poor (*n* = 15), respectively.

#### Workforce aspects

**FIGURE 5:**
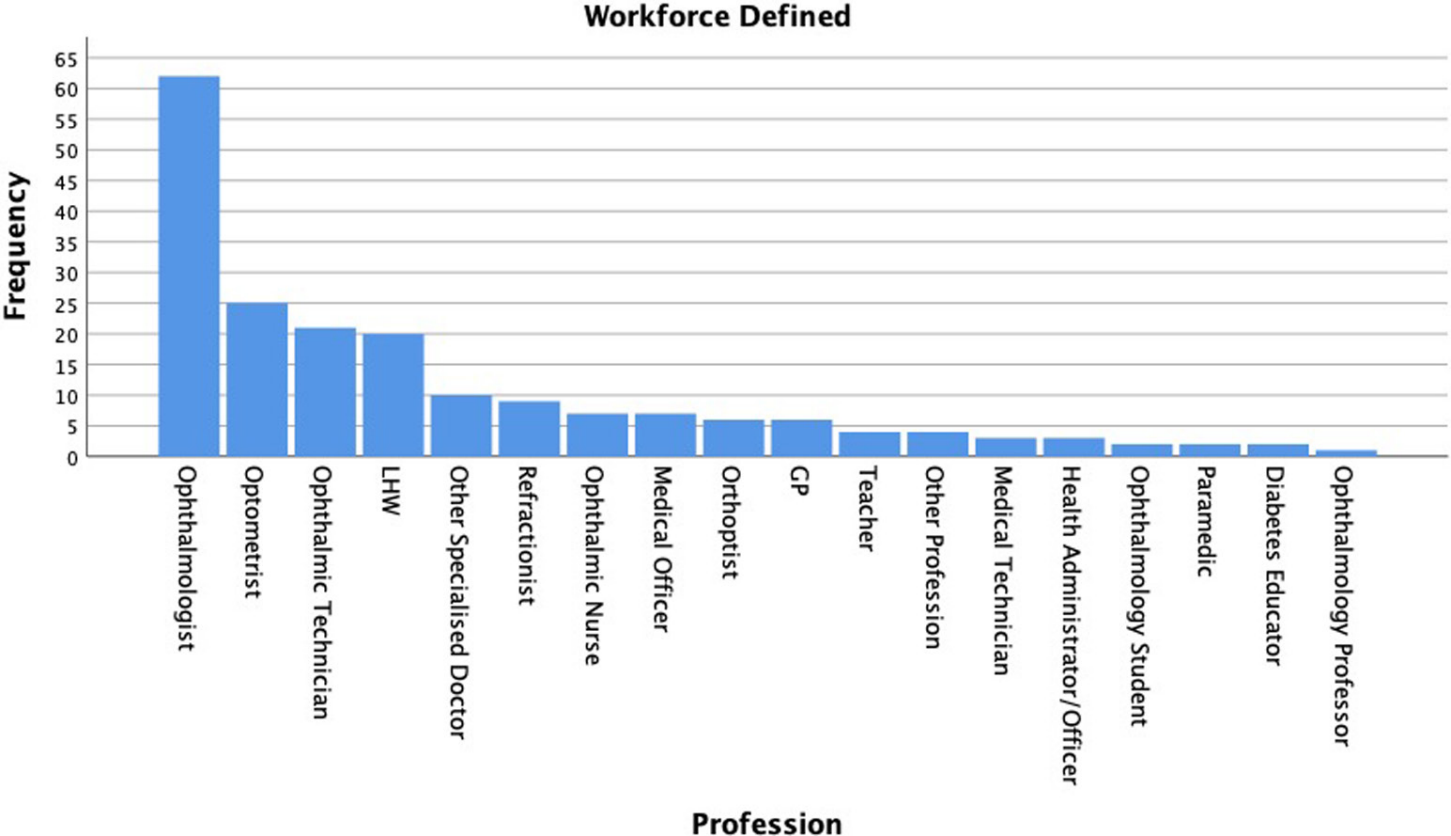
Bar chart showing the frequency of different professions which were mentioned across 79 papers. The category ‘Other Specialised Doctor’ includes oncologists, diabetologists, paediatricians, neonatologists and endocrinologists. The category ‘other profession’ includes a statistician, histopathologist, social worker and counsellor. LHW: Lady health worker; GP: General medical practitioner

Out of 132 papers, 60% (*n* = 79) defined a specific workforce to some capacity, with 41% (*n* = 54) mentioning more than one profession. The frequency of the different workforces which were defined are listed in Figure 5 below. Ophthalmologists were mentioned most often (*n* = 62), followed by optometrists (*n* = 25), ophthalmic technicians (*n* = 21) and Lady Health Workers (LHW) (*n* = 20).

#### Age of population

**FIGURE 6:**
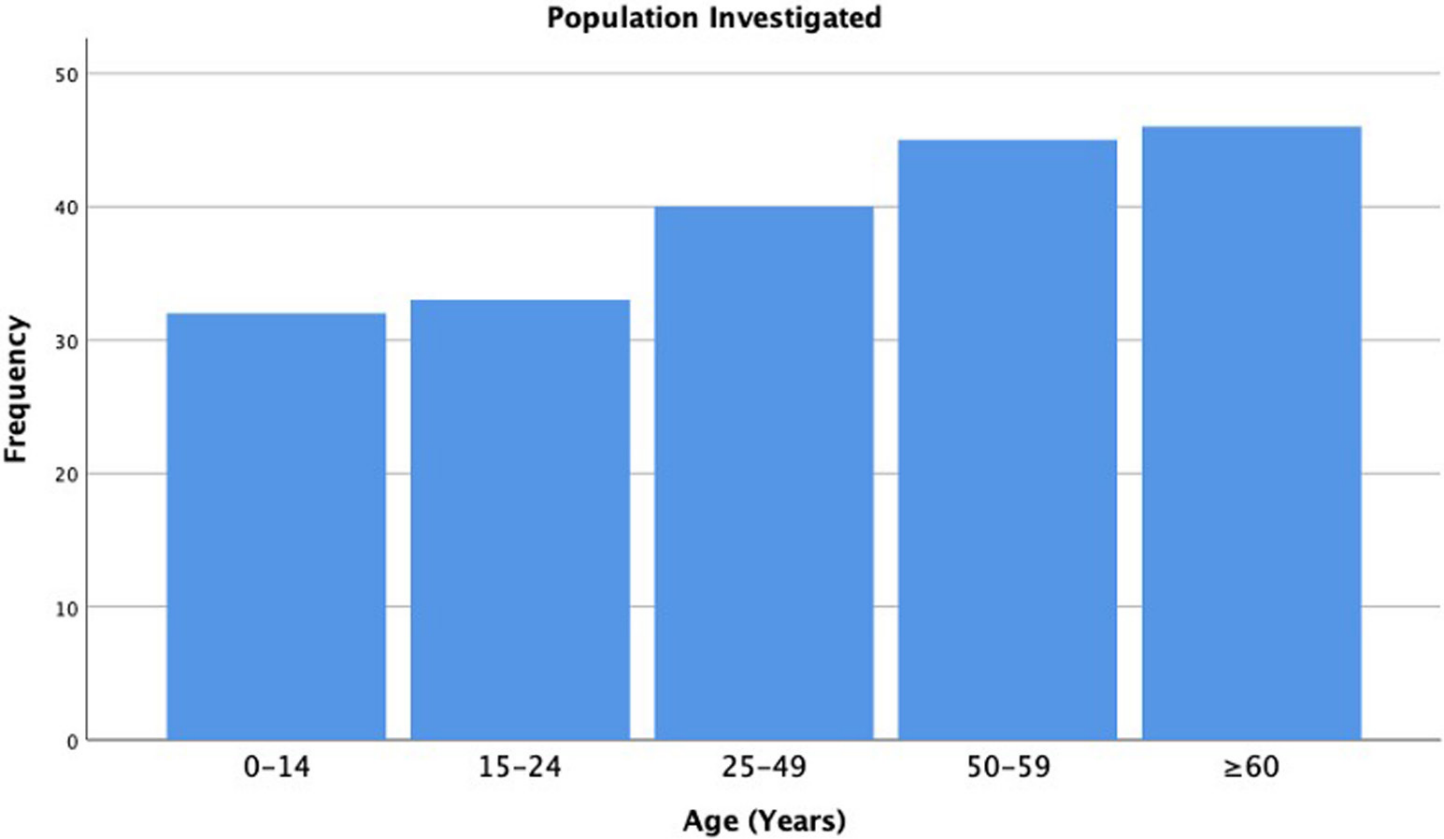
Bar chart showing frequency of age groups investigated across 71 papers

Out of 132 papers, 81 recorded the age group(s) under investigation. There was significant overlap between age groups investigated within papers. Only 10 papers mentioned an age group that did not overlap with another, and 10 papers covered all age groups and were therefore not included in the final result. Out of the remaining 51/132 papers, 12 did not record which age group the paper investigated and for 39 papers the age group was not applicable to the study design. Figure 6 shows the distribution of papers across the different age groups. As the age group increases the number of papers carried out also increases. Although the majority (*n* = 46) of papers covered the >60‐year age group, only two papers focused solely on this age group.^82^, ^105^

#### Thematic analysis

**FIGURE 7:**
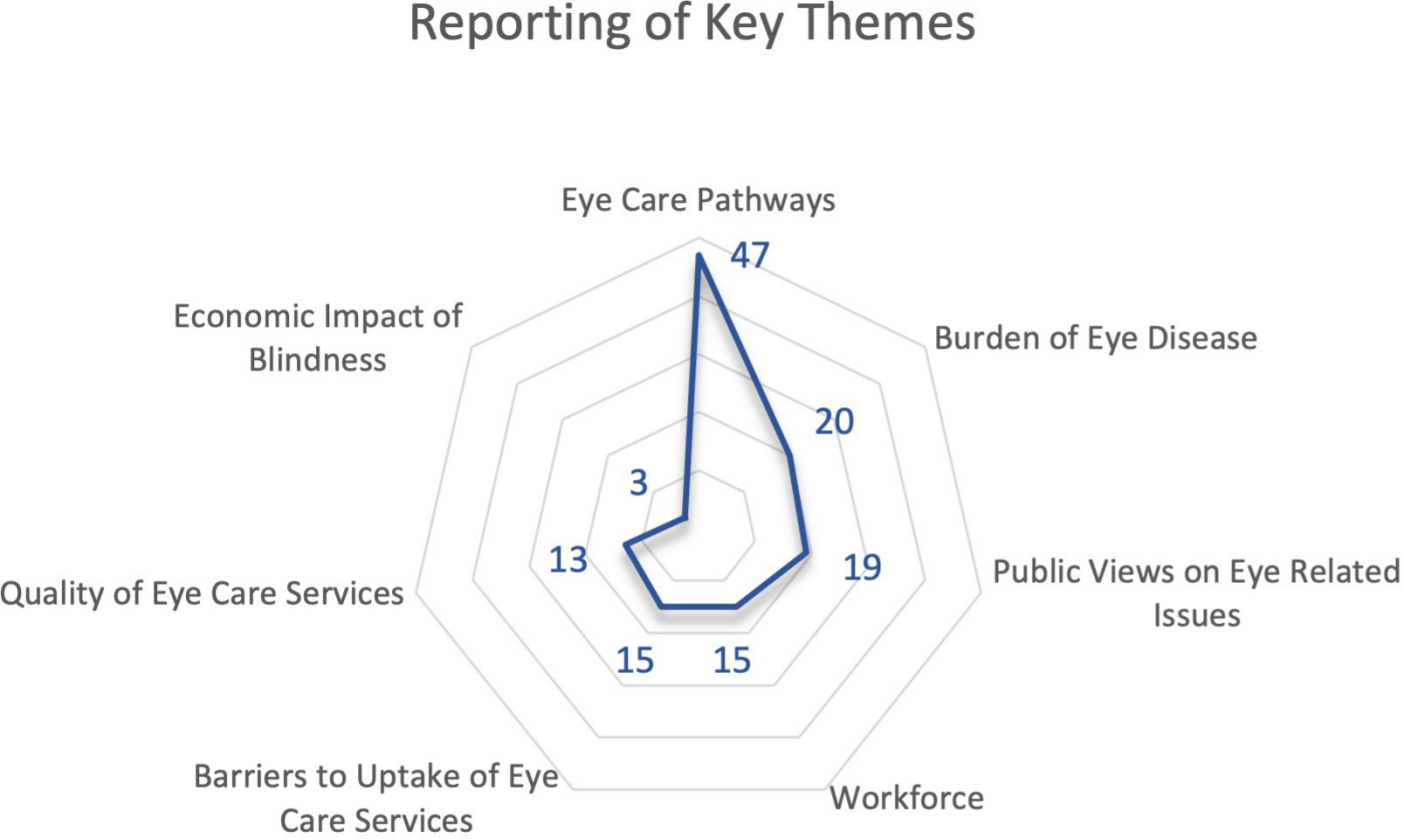
Radar chart showing seven major themes which were identified, and the frequency at which they were reported across 132 papers

Papers were grouped according to their topic subject. A total of 11 topics were identified, comprising specific ophthalmic conditions as well as wider aspects of eye care, including cataract, cornea, prevention of blindness plans, DR, gender inequality in eye care, glaucoma, ocular injuries, paediatrics, workforce, prevalence studies and poverty associated with vision loss. The papers were then coded resulting in seven primary themes, and displayed in a radar chart to illustrate the unequal distribution of themes identified graphically (Figure 7). The most common themes are listed as follows: eye care pathways (36%, *n* = 47), burden of eye disease (15%, *n* = 20) and public views on eye related issues (14%, *n* = 19). No new themes were identified from the consultation exercises. For each theme, the results of the scoping review are discussed. Table 3 provides a summary of the key themes and additional input from the stakeholder consultations.

**TABLE 3:** Summary of key themes organised by subthemes, with relevant examples and data collected from the consultation exercises, along with an average quality rating score ± SD for papers under each theme

Primary theme	Subthemes identified within the key theme domain	Number of papers^(refs)^	Example	Additional data from expert consultation	Average quality rating (SD)
Eye care pathways (*n* = 47)	Screening programmes	21^(^ ^108^, ^109^, ^110^, ^112^, ^114^, ^115^, ^116^, ^117^, ^119^, ^127^, ^128^, ^129^, ^130^, ^132^, ^133^, ^135^, ^136^, ^138^, ^139^, ^140^, ^141^ ^)^	Screening services have been used to examine ocular health and refractive errors amongst school children.^109^, ^116^, ^129^, ^133^, ^136^, ^138^, ^140^ Attempts have also been made to provide eye care through the indigenous educational system in Pakistan: the madaris.^110^, ^127^, ^146^	District comprehensive eye care services and screening programmes in Pakistan are largely being implemented in the Punjab province, when compared with the remaining six provinces.	0.60 (0.13)
	Management of chronic eye conditions	10^(^ ^6^, ^111^, ^119^, ^120^, ^125^, ^128^, ^131^, ^135^, ^139^, ^151^ ^)^	The frequency of DR in a military hospital (28.67%), was lower than that reported in other studies. One reason given was that medical facilities set up by the army are more organised, free and easily available to those accessing them.^111^		
	Referral systems	6^(^ ^113^, ^121^, ^124^, ^126^, ^128^, ^149^ ^)^	A qualitative evaluation into the referral system for retinopathy of prematurity (RoP) showed that the majority of participating centres did not have any referral system for patients diagnosed with RoP.^149^ Out of the two centres who did have a referral pathway, the protocols in place regarding referral were not being followed.		
	Trachoma control	5^(^ ^6^, ^118^, ^120^, ^131^, ^151^ ^)^	A decrease in the frequency of trachoma has been noted in areas where provision of better‐quality water sources to communities was observed leading to improved sanitation levels.^6^		
Burden of Eye Disease (*n* = 20)	Blindness and VI	8^(^ ^9^, ^14^, ^25^, ^94^, ^98^, ^102^, ^103^, ^104^ ^)^	The main causes of blindness in Pakistan were cataract and refractive error.^9^, ^14^, ^102^, ^104^	One stakeholder with extensive expertise in clinical vision research, identified the additional unmet need for prevalence research and statistics on myopia and dry eye disease in Pakistan.	0.60 (0.08)
	Anterior eye conditions	6^(^ ^28^, ^92^, ^93^, ^95^, ^96^, ^101^ ^)^	The prevalence of stromal corneal dystrophies in hospitals in Lahore was 0.4%.^93^		
	Diabetic retinopathy	4^(^ ^26^, ^97^, ^99^, ^100^ ^)^	A systematic review^26^ of 29 studies reported a prevalence for DR of 28.8% with considerable variation between 10.6% to 91.3%.		
	Age‐related macular degeneration	1^(^ ^105^ ^)^	The prevalence of age‐related macular degeneration was 1.6% in a tertiary care hospital in Lahore.		
	Retinoblastoma	1^(^ ^106^ ^)^	Pakistan has been shown to have the 4th highest incidence of retinoblastoma in the Asia‐Pacific region.		
Public Views on Eye Related Issues (*n* = 19)	Awareness and views of eye related issues affecting children	6^(^ ^74^, ^76^, ^78^, ^84^, ^88^, ^91^ ^)^	A study carried out by Latif et al.^84^ found that 72.4% of their participants (children and their parents) had never visited an ophthalmic service for a clinical examination.	One subject expert suggested local optometrists should provide visual task analysis for local industries (i.e., welding, cement factories) and educate the employers and employees on ocular safety and visual hazards.	0.66 (0.15)
	Awareness of eye related issues amongst the general population and common misconceptions	5^(^ ^82^, ^85^, ^86^, ^87^, ^91^ ^)^	A study carried out in the rural population of Sahiwal revealed a poor level of knowledge and awareness of glaucoma and its risk factors.^85^ All participants had heard about glaucoma, although misconceptions included that it was another name for cataract (25%) and damage caused by it was reversible (75.6%).		
	Public knowledge surrounding ocular safety	4^(^ ^74^, ^77^, ^83^, ^89^ ^)^	Hassan et al.^77^ assessed the level of awareness of occupational health hazards amongst welders in Lahore; 45.7% believed that there were no serious health risks involved with welding. Similar awareness levels were noted amongst women cotton workers at risk of pesticide exposure in southern Pakistan; 55% of respondents were not using any protective measure during cotton picking.^74^		
	Patient experiences with eye care services (i.e., cataract surgical services)	4^(^ ^73^, ^79^, ^80^, ^81^ ^)^	Patient experiences of visual sensations during cataract surgery in Pakistan were explored, and limited pre‐operative counselling was reported.^80^, ^81^		
Workforce (*n* = 15)	Task‐sharing	8^(^ ^58^, ^59^, ^60^, ^61^, ^62^, ^64^, ^65^, ^70^ ^)^	An example of successful task sharing was recorded by Shaheen and Khan,^70^ who studied optometric services in tertiary hospitals in Lahore following the introduction of optometrists in out‐patient departments. A key outcome of the study was that 60% of ophthalmologists felt their workload had decreased since working with optometrists and the quality of work had improved.	The most common concern raised was the non‐existent regulatory framework and body for the optometric profession. This led to discussions about the potential lack of uniformity in clinical standards, accompanied by apparent differences in training programmes across the country, different levels of responsibility for optometrists depending on the province, and lack of awareness from other health professionals about the role of an optometrist. Conflict also appears to exist within the optometric profession, where different levels of education and training curricula result in a variable knowledge base amongst practitioners with the same job title. With regards to involvement of LHWs and teachers in eye care, consultations indicated that their contribution to eye care is perceived as fairly minimal. While some local projects involved these cadres in detection of visual problems: ‘…they only worked well in the short term.’ (Optometrist, Punjab).	0.56 (0.13)
	Training programmes (i.e., training teachers and lady health care workers in eye care)	5^(^ ^63^, ^65^, ^66^, ^67^, ^72^ ^)^	A comparative evaluation of knowledge of primary eye care in trained versus untrained teachers in Pakistan showed that both groups of teachers had good knowledge about eye health, however untrained teachers were more likely to yield to misconceptions.^63^		
Barriers to the uptake of eye care services (*n* = 15)	Gender disparity	5 ^(^ ^8^, ^43^, ^45^, ^47^, ^53^ ^)^	A study^47^ carried out in a tribal area in KPK found that women had a 2.1‐fold greater prevalence of bilateral cataract blindness than men and cataract surgical rates were also lower for women (41.3% for women versus 46.0%–49.5% for men).	Introduction of the Sehat Sahulat Programme (SSP), a micro health insurance scheme. The SSP provides expenditure for health care to people living below the poverty line (earning <$2 a day). While it is still being rolled out across the country, one participant noted that it is helping to reduce cost as a barrier to uptake of eye care services.	0.75 (0.13)
	Accessibility (i.e., being able to get to the clinic)	5^(^ ^8^, ^23^, ^46^, ^50^, ^52^ ^)^	Lack of reliable transport options and poor roads cause difficulty when trying to travel to eye care facilities.^46^		
	Cost	6^(^ ^24^, ^44^, ^47^, ^48^, ^50^, ^51^ ^)^	Cost of cataract surgery has been a major barrier in preventing individuals from undergoing cataract surgery.^47^, ^48^, ^51^		
	Literacy levels & public knowledge	4^(^ ^8^, ^44^, ^49^, ^54^ ^)^	A survey^8^ found that subjects who had attended primary school were 60% less likely to have a VA <6/60 (legal blindness) than subjects who had never been to school. A cross‐sectional study in Punjab found that lack of knowledge was a major barrier to compliance with regular DR screening.^49^		
Quality of Eye Care Services (*n* = 13)	Quality of cataract surgical services	9^(^ ^30^, ^31^, ^32^, ^34^, ^35^, ^36^, ^40^, ^41^, ^42^ ^)^	Recent literature identified from the search^31^, ^35^ has shown the rate of cataract surgery with an IOL has increased in Pakistan compared with earlier studies.^32^, ^41^	Stakeholders confirmed and emphasised variability in quality of services across the country. Adding that surgical rates and quality of services are generally better amongst urban populations in the Punjab, Sindh and KPK provinces and are of greater quality in the private sector for paying patients. ‘Punjab is the most settled province in Pakistan so there you will get good facilities.’ (Optometrist, KPK). Reasons given for these variations included that practitioners with the same job title provide a varying level of care in Pakistan as their training is not uniform across the country, adversely affecting the quality of eye services provided.	0.71 (0.12)
	Quality of services varying based on location and gender	4^(^ ^29^, ^34^, ^40^, ^41^ ^)^	Ahmad et al.^34^ found that 15.2% of participants in a marginalised marine fishing community in Karachi had undergone cataract surgery, 91.7% with an IOL. However, one quarter of the surgeries resulted in patient dissatisfaction with two thirds of 145 eyes resulting in some form of visual loss and 12.4% blind after operation; women also experienced substantially worse visual outcomes compared with men.		
Economic Impact of Blindness (*n* = 3)	Financial benefit from investing in eye care	1^(^ ^55^ ^)^	Treating avoidable blindness in Pakistan can result in an economic benefit of US$4.25 billion over a ten‐year period, as this frees up carers to contribute to mainstream economic activity.	Provincial funding for eye care provided by the government was known amongst stakeholders working in Pakistan; however they alluded that in some provinces these funds are not solely dedicated to eye care as intended according to local eye health plans.	0.70 (0.01)
	Financial support required to sustain and deliver eye care services	1^(^ ^56^ ^)^	Costs of diabetic and DR screening and detection in the community (through eye camps) and in a hospital setting were compared. The cost per person screened in the community was shown to be lower due to the high volume of patients screened through eye camps. However, the cost per diabetic diagnosis and diabetic retinopathy identified was higher in a community setting compared to a hospital setting.		

Abbreviation: KPK, Khyber Pakhtunkhwa province.

#### Theme 1: Eye care pathways

The theme of eye care pathways encompassed 38 studies^6^, ^108^, ^109^, ^110^, ^111^, ^112^, ^113^, ^114^, ^115^, ^116^, ^117^, ^118^, ^119^, ^120^, ^121^, ^122^, ^123^, ^124^, ^125^, ^126^, ^127^, ^128^, ^129^, ^130^, ^131^, ^132^, ^133^, ^134^, ^135^, ^136^, ^137^, ^138^, ^139^, ^140^, ^141^, ^142^, ^144^, ^149^ and 9 other relevant documents^15^, ^107^, ^143^, ^145^, ^146^, ^147^, ^148^, ^150^, ^151^ and overlaped considerably with the other themes. This theme directly relates back to our research question where we aim to explore the evidence surrounding eye care pathways in Pakistan. Pakistan's public eye health services largely resemble district comprehensive eye care services (DCECS) which were created following a situation analysis to identify barriers to the uptake of cataract surgery at a district level.^107^ DCECS have shown great success in many districts across Pakistan, with an increase in outpatient attendance rates and cataract operations.^15^, ^107^, ^122^

The majority of studies under this theme reported on screening programmes.^108^, ^109^, ^110^, ^112^, ^114^, ^115^, ^116^, ^117^, ^119^, ^127^, ^128^, ^129^, ^130^, ^132^, ^134^, ^135^, ^136^, ^138^, ^139^, ^140^, ^141^ Papers reporting on the prevalence of DR in Pakistan have expressed the need for organised and consistent DR screening programmes.^111^, ^112^, ^114^, ^121^, ^125^, ^128^, ^130^, ^135^, ^143^, ^151^ Other screening services available in the country include school screening^109^, ^116^, ^129^, ^133^, ^136^, ^138^, ^139^ and screening for cataracts through the use of eye camps.^117^ Ten papers discussed the management of chronic eye conditions including DR,^111^, ^125^, ^128^, ^135^ glaucoma^119^, ^139^ and trachoma.^6^, ^120^, ^131^, ^151^ Six studies discussed referral systems.^111^, ^119^, ^122^, ^124^, ^126^, ^147^ Five papers focussed on trachoma control.^6^, ^118^, ^120^, ^131^, ^151^

#### Theme 2: Burden of eye disease

Twenty studies^9^, ^14^, ^25^, ^26^, ^28^, ^92^, ^93^, ^94^, ^95^, ^96^, ^97^, ^98^, ^99^, ^100^, ^101^, ^102^, ^103^, ^104^, ^105^, ^106^ focussed solely on the epidemiology of eye conditions and the prevalence of blindness in Pakistan. The results from these studies proved useful in establishing a summative description of eye care in Pakistan as they highlight the magnitude of the demand for eye care in the country. The largest subgroup within this theme (*n* = 8) reported on blindness or refractive error.^9^, ^14^, ^25^, ^94^, ^98^, ^102^, ^103^, ^104^ Dineen et al.^9^ found that refractive error causing severe visual impairment was more common amongst rural dwellers. They also estimated that 904,000 adults in Pakistan had an operable cataract causing a visual acuity of 6/60.

Epidemiology papers covering a variety of anterior eye conditions have also been undertaken, covering corneal dystrophies/ulcers, keratoconus, uveitis and trichiasis.^28^, ^92^, ^93^, ^95^, ^96^, ^101^ A national trachoma programme has been implemented in Pakistan, adopting the WHO SAFE strategy to control the disease. The successful strengthening control of trachoma in Pakistan is reflected in a major achievement, namely the drastically reduced prevalence of trichiasis estimated by Flueckiger et al.^96^: 2009: 71,700 cases; 2012: 23,420 cases; 2015: 5330 cases; reducing the prevalence by more than 90% of original estimates over a 6‐year period.

#### Theme 3: Public views on eye related issues

Patient priorities in terms of eye care are largely explored under themes 3 and 5. Nineteen studies^73^, ^74^, ^75^, ^76^, ^77^, ^78^, ^79^, ^80^, ^81^, ^82^, ^83^, ^84^, ^85^, ^86^, ^87^, ^88^, ^89^, ^90^, ^91^ in this theme reported on public views on eye care. Public views and experiences of cataract surgery have been assessed.^73^, ^79^, ^80^, ^81^ The research showed that there are misconceptions surrounding cataract surgery amongst the public. Abdullah and Abdullah^82^ revealed that some participants wrongly thought that the intra‐ocular lens (IOL) was required to be replaced every year, which led to hesitation in undergoing surgery, largely due to concerns about long‐term follow‐on costs. Another deterrent was if family members had experienced unsuccessful operations.

Six studies explored the knowledge, attitude and practices relating to children's eye health.^74^, ^76^, ^78^, ^84^, ^88^, ^91^ Five studies^82^, ^85^, ^86^, ^87^, ^90^ also evaluated public knowledge and awareness surrounding other eye conditions including glaucoma.^85^ The public's awareness of ocular safety has also been discussed in studies pertaining to the welding profession^77^ and female cotton workers.^74^

#### Theme 4: Workforce

Sufficient human resources are pertinent in maintaining a stable eye care pathway, and the growing demand on eye care services in Pakistan has raised a need to evaluate the workforce.^71^ Re‐structuring of training programmes for optometrists and ophthalmologists have been discussed;^69^, ^72^ however, task‐sharing appears to be timely and topical.^58^, ^59^, ^60^, ^61^ In a series of interviews, one of the few qualitative studies identified that factors which favour task sharing included a high demand for eye care services and a preference of the name “task sharing” rather than “task shifting”.^59^ Barriers to implementation of task‐sharing were also explored. Jamal and Chaudhry^64^ noted potentially conflicting interests affecting the realisation of task sharing, i.e., resistance from some ophthalmologists regarding co‐management in glaucoma and disease diagnosis. Shah et al.^59^ noted a lack of human resources and coordination amongst health professionals and policy makers as a major barrier.

Another group of eye care personnel which have been used to deliver healthcare to women and promote eye care awareness are LHWs.^67^, ^68^ This professional group plays a vital role in primary health care by offering immunisation, maternity, child and family planning services. Each LHW visits 5–7 households a day. A new eye care curriculum was piloted amongst LHWs in the Federally Administered Tribal Area from 2001 to 2005.^66^ This training resulted in a positive outcome with LHWs more involved in primary eye care by providing an increased amount of vision screening and appropriate referrals when compared with colleagues in other provinces who had not received the training. Surprisingly and innovatively, schoolteachers are also being trained in primary eye care.^63^

#### Theme 5: Barriers to uptake of eye care services

Twelve studies^8^, ^24^, ^27^, ^45^, ^46^, ^47^, ^48^, ^49^, ^50^, ^51^, ^52^, ^54^ and three other relevant documents^43^, ^44^, ^53^ identified barriers to the uptake of eye care services. Of these, a third reported gender disparity within the population as a major barrier to accessing services.^8^, ^43^, ^45^, ^47^, ^53^ Five studies identified challenges to access services as a major barrier.^8^, ^23^, ^46^, ^50^, ^52^ For example, it was reported that people living in rural areas needed to travel long distances to access eye care provisions.^46^ Six papers identified the cost of healthcare services as negatively impacting upon people's ability to access care.^24^, ^44^, ^47^, ^48^, ^50^, ^51^ Some identified a number of facilitators to overcome many of these challenges, including free transport and widening access by increasing opening hours and subsidising services.^43^, ^44^, ^53^ Four papers also identified literacy levels and public knowledge as a further barrier to the uptake of eye care services.^8^, ^44^, ^49^, ^54^ Low literacy levels were associated with an increased prevalence of visual impairment and blindness.^8^, ^54^

#### Theme 6: Quality of eye care services

Amongst the 13 papers^30^, ^31^, ^32^, ^33^, ^34^, ^35^, ^36^, ^37^, ^38^, ^39^, ^40^, ^41^, ^42^ that reported on quality of eye care, nine studies^30^, ^31^, ^32^, ^34^, ^35^, ^36^, ^40^, ^41^, ^42^ discussed the quality of cataract surgery in Pakistan. Outcome measures of quality included cataract surgical outcomes and whether an IOL was used versus leaving the patient aphakic postoperatively. The studies showed that patients who underwent cataract surgery with an IOL had better visual acuity following refraction than patients receiving non‐IOL surgery.^31^, ^32^, ^35^ The papers also provided evidence that the quality and availability of cataract services appears to vary between urban and rural settings, with a greater prevalence of blindness as a result of unoperated cataract and uncorrected aphakia in rural areas.^40^ The Pakistan National Blindness and Visual Impairment Survey^41^ also found that eye camp surgery, rural residence and female sex were more likely to result in a visual acuity of <6/18 (i.e., less than the standard for driving in many countries).

#### Theme 7: Economic impact of blindness

This theme encompassed three papers.^55^, ^56^, ^57^ One study estimated the economic benefits associated with rehabilitating the blind population of Pakistan, which allows carers to resume participation in mainstream economic activity.^55^ Another study carried out in Rawalpindi compared the costs of diabetic and DR screening and detection in the community (through eye camps) and in a hospital setting.^56^ Finally, a case study reported on funding secured from the government of Pakistan for a sum of US$51 million to support the third National Plan for Prevention of Blindness (2005–2010), although it did not provide details on how the funds would be utilised or reasons justifying the figure allocated to the plan.^57^

#### Additional input from expert stakeholders

The review's findings were considered comprehensive by all stakeholders in terms of their understanding and coverage of the literature field surrounding eye care in the country. The consultations also indicated current limitations of eye care services in Pakistan, and opportunities for further improvements of the eye care model through advancement of the eye care workforce and increasing accessibility to services in rural areas (see Table 3). Stakeholders pointed out that common eye conditions such as progressive myopia and dry eye are not yet research priorities, as basic eye care needs are not fully met equitably in all parts of the country and across all population groups. One stakeholder working as an optometrist in the Punjab province also highlighted the rollout of the Sehat Sahulat programme, a micro health insurance scheme, as a facilitator for people living below the poverty line to seek treatment for their cataracts. Funding provided by the government to implement national plans for the prevention of blindness were well known amongst stakeholders working in Pakistan. One participant voiced concerns regarding lack of audits in the ophthalmology department stating that “they are providing costly things and supporting me economically, but no one is checking how I am using these things.” Therefore, the participant stressed the need for economic evaluations and audits of eye care services.

**FIGURE 8:**
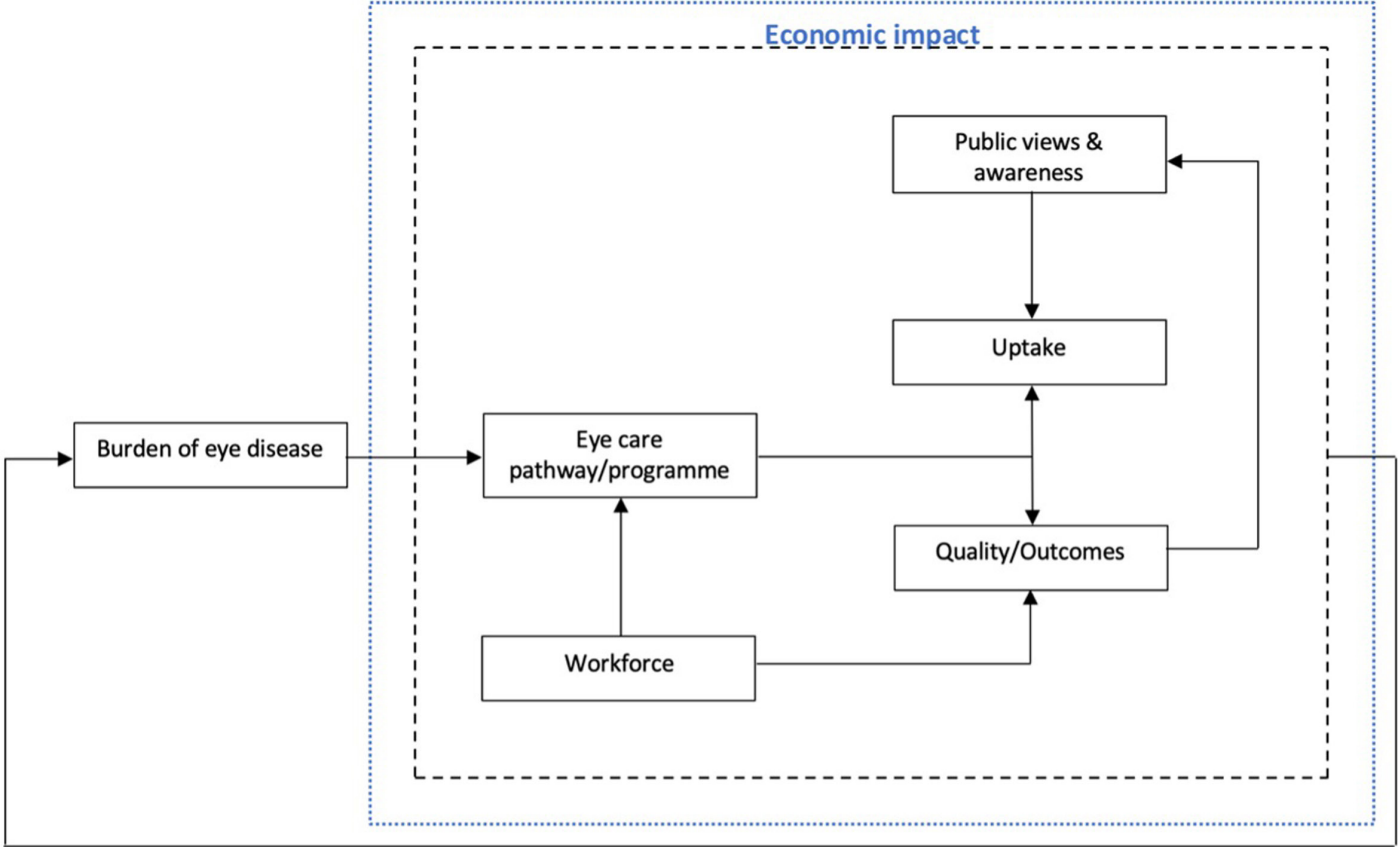
Framework showing relationships between seven themes identified from the scoping review. The theme economic impact of blindness is directly related to the five themes encompassed within the black dotted line. These six themes feedback and affect the burden of eye disease theme

**FIGURE 9:**
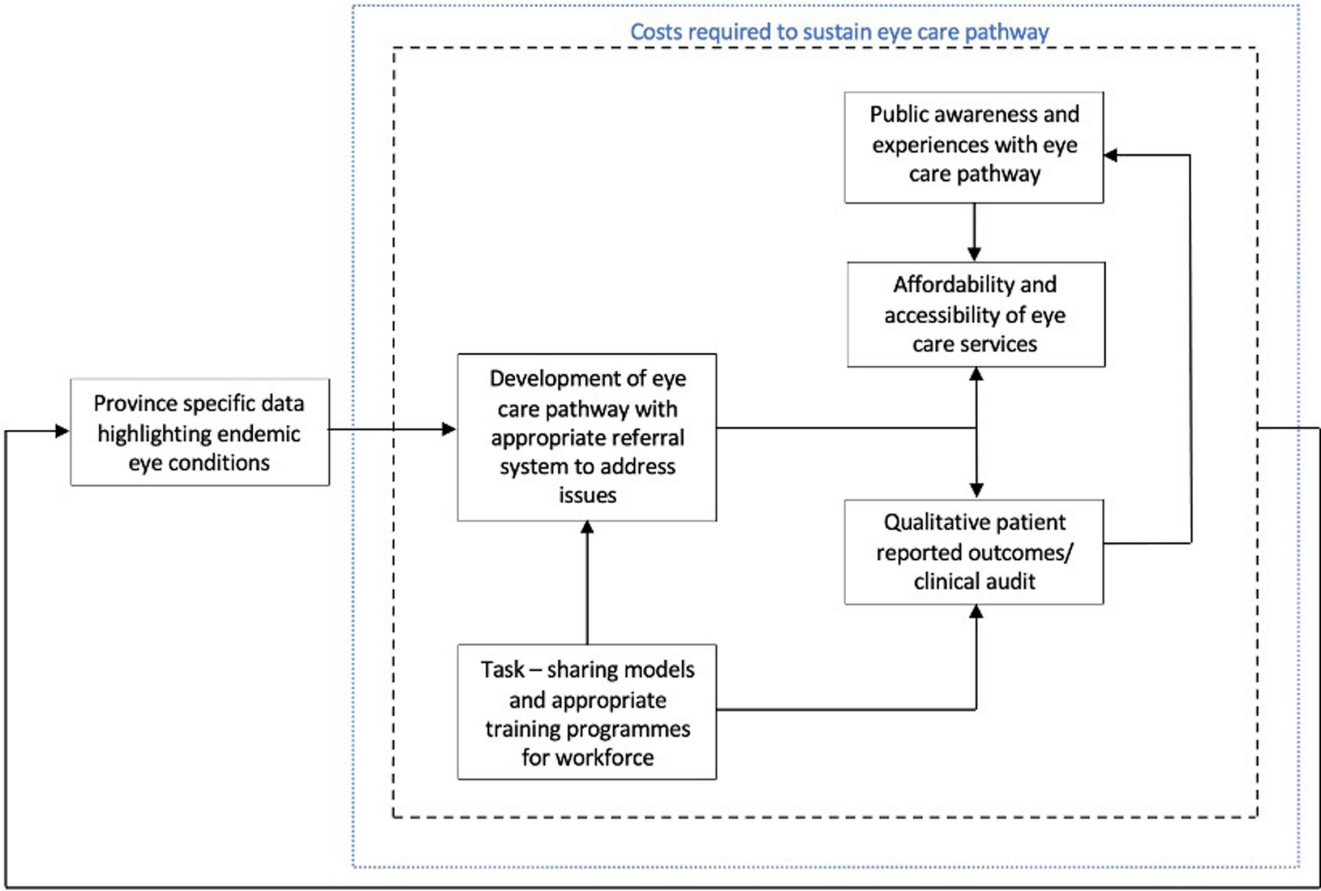
Research priorities identified from scoping review substituted into the framework outlined in Figure 8. Priorities within the black dotted line are influenced directly and/or indirectly by associated costs and finances, which have an effect on the eye care pathway's long‐term sustainability. The prevalence of endemic eye conditions is influenced by all six priorities

Following the expert consultations, it became evident that many of the themes identified must be considered in parallel, as they are clearly interrelated. To illustrate the links, Figure 8 shows the relationship between the different themes. The theme of burden of eye disease is required to identify problems, their size and location. This information provides a rationale to developing an eye care improvement programme to target the problem. The eye care programme/pathway is dependent upon the workforce supporting it. The success of the eye care programme is then dependent on the uptake (patient awareness and views also affect this) and the outcomes/quality of its services (also influenced by workforce aspects). The sustainability of the pathway/programme is reliant upon the associated costs and finances required. All these aspects combined create a feedback loop to the burden of eye disease as their status affects the prevalence of eye conditions in the country e.g., a strong workforce improves outcomes of the eye care programme through increased examination rates, which results in a decrease in avoidable blindness. Figure 9 combines gaps in literature with research priorities identified during consultations and substitutes them into the framework outlined in Figure 8, highlighting research priorities and an approach to investigate them.

## DISCUSSION

This review identified 132 papers reporting on eye care in Pakistan. The overall quality of existing research within this field was good, but variable, with about half of all included studies (*n* = 60) achieving a quality score of <0.70 (poor‐adequate quality). This review also indicates that previous research on eye care in Pakistan contains relatively little empirical qualitative evidence, which is becoming an important part of health research when displaying views and experiences of patients. Additionally, this review highlights that the majority of papers investigated issues in only three provinces, Punjab, Sindh and KPK. These areas have the benefit of being more economically advanced and stable, with a larger population density than the other provinces in Pakistan. The majority of the larger educational institutions offering programmes and degrees related to ophthalmics are situated in these three provinces, resulting in other provinces being infrequently considered in papers. While the findings of our consultation exercise provide a snapshot of our stakeholders' perspectives, there remains a possibility that other stakeholders, e.g., in other provinces where we did have not have access, may have either complementing or contrasting perspectives on this.

National and provincial co‐ordinators for eye health from the Ministry of Health in Pakistan were also invited to participate in the consultation exercise. However, despite our efforts we were not able to recruit a current member. Nonetheless, an experienced former national co‐ordinator for eye health in Pakistan participated, and provided feedback on our results including information gathered from the consultation exercise. The former co‐ordinator largely agreed with the results of our review and the comments made by the stakeholders. In terms of geographical distribution of papers, the co‐ordinator was surprised by the lack of papers from KPK, as this province hosts the Pakistan Institute of Community Ophthalmology (PICO) and suggested some papers may be missing or not easily accessible. When DCECS were discussed, two reasons arose as to why they are working better in Punjab when compared with other provinces. The first reason was that the current national co‐ordinator for eye health is based in Punjab, and therefore more efforts are focussed within this province. The second reason given was that the provincial government in Punjab is more responsive than others when addressing the needs of its province. A lack of infrastructure and human resources were reasons given for the lack of research in underserved provinces such as Balochistan, Gilgit‐Baltistan and Azad Jammu and Kashmir.

Our scoping review aimed to identify the volume and scope of current evidence around three core areas: pathways, patient priorities and economics relating to eye care in Pakistan. Thematic analysis of the final search results produced seven themes mapped to the research question. The themes eye care pathways, quality of eye care services, workforce influencing existing pathways and burden of eye disease leading to the creation of certain pathways (i.e., to target trachoma control), all relate back to ‘pathways’ in our initial research question. The most common themes identified were eye care pathways, followed by burden of eye disease, with DR prevalence studies thoroughly reviewed. Additionally, there was a lack of research surrounding the quality of eye care facilities in Pakistan and the workforce currently providing eye care services, limiting the opportunities for developing robust, evidence‐based policies. Patient priorities were addressed under the themes: barriers to uptake of eye care services and public views on eye related issues. Teacher and parental perception of eye conditions and disproportionate access to eye care services for females were areas of research that received the most attention. However, methods to improve access to eye care services for rural populations, e.g., through the use of tele‐ophthalmology, were limited. The theme economic impact of blindness summarises the literature available surrounding the economic aspect of eye care. Papers were scarce (*n* = 3) with regard to the economic aspects of blindness, but the quality of those studies was generally good.

There was also a scarcity of evidence on older populations, with only two papers focussing on the over 60‐year age group.^82^, ^105^ These findings suggest that the elderly category is typically grouped together with the working age group, indicating that there is potential to concentrate explicitly on this age group (≥60 years) in the future, as their expectations and experiences of eye care services may differ. Importantly, their (the elderly) eye and health care needs may differ from those of working‐age populations, as they are more likely to have age‐related conditions including cataract, glaucoma and macular degeneration. Elderly populations are also likely to face a different set of challenges, such as mobility issues, which could result in a high dependence on the working‐age population to access care and a reliance on financial resources to pay for care.

### Implications for future research

After synthesising the outcomes of the scoping review and subsequent expert consultation, we have identified key areas for future research. Consistency amongst the workforce and training programmes would be beneficial, with optional implementation of task‐sharing models to reduce the immense and increasing workload placed on ophthalmologists in the country. Furthermore, the quality of current services provided should be evaluated. To give patients a voice, patient experiences utilising eye care services should be investigated, and methods for improvement of current eye care services should be explored. Evaluation of eye care services available in rural areas is also a priority. Finally, evaluation of the costs and benefits related to eye care provision and in sustaining eye care programmes is another key area requiring research.

### Strengths and limitations

The strengths of the review include the broad approach taken as it is the first of its kind to comprehensively identify, analyse and synthesise quantitatively, as well as qualitatively, all literature published and unpublished, concerning the topic of eye care in Pakistan. The addition of consultations with expert stakeholders provided further valuable insight, indirectly validating the results from the scoping review, as participants described real examples and lived real‐world experiences related to the challenges present with eye care service and delivery in the country. Applying a quality assessment and critical appraisal of included studies allowed for a first evaluation of the robustness of the existing evidence.

While using a broad and inclusive approach in this review, scoping reviews have limitations. These include the lack of assessment for bias, as they typically do not incorporate quality appraisal.^152^ However, the objective was to obtain a comprehensive insight into eye care in Pakistan and a scoping review arguably is the tool best suited for this purpose. There is also an ongoing debate surrounding whether quality appraisal of included papers should be carried out in a scoping review.^17^, ^152^, ^153^ In this review a detailed and multifaceted quality appraisal of all included studies was undertaken in order to identify the nature of research in each area and to ensure methodological rigour. The “QualSyst” tool was chosen to evaluate the quality of the included studies, in line with other systematic reviews which evaluated studies with multiple study designs.^154^, ^155^, ^156^ However, a perceived limitation of QualSyst is the relatively restricted sensitivity which may occasionally result in somewhat inflated quality grades.^154^, ^156^ This limitation was mitigated by adopting thresholds defined in previous studies.^154^, ^155^, ^156^ While this approach to quality assessment is to be interpreted with caution, it provides: (i) an initial indication of study quality and (ii) a tool to allow comparison of different studies on the same theme.

### Implications for clinical practice and eye care policy internationally

The review revealed that currently there is not a cohesive eye care pathway in Pakistan, but considerable fragmentation of eye care within the country. Potentially, the frameworks detailed in Figures 8 and 9 can form the basis of an evaluation tool to assess the viability of eye care services in Pakistan. This would aid in strengthening existing or creating new eye care services and addressing eye care, blindness and VI as WHO priority areas.^1^ It also aligns with the WHO framework for integrated people‐centred eye care (IPEC)^3^ as the framework considers the availability, accessibility and performance of services offered by the eye care workforce. Pakistan, a member state of the WHO, is also aligning with the IPEC framework in an attempt to work towards providing universal health care. The WHO have also developed an eye care assessment tool^158^ (ECSAT) comprising a detailed checklist based on the WHO health system approach framework,^159^ which organises health systems around six fundamental components or “building blocks”: leadership and governance, financing, the health workforce, service delivery, access to vital medicines and health information systems. The ECSAT tool gives an overview of the primary gaps, requirements, and information requiring further research as well as advised measures to address these issues within a country.^166^ Whereas our framework allows for the evaluation of existing services as well as the creation of new sustainable eye care services through the incorporation of research priorities (Figure 9).

Previous studies surrounding access to eye care have been criticised for failing to investigate linkages between eye care services and their outcomes, or between service use and patient satisfaction.^160^ Our framework engages people and communities through the exploration of their views and experiences with eye care systems, considering patient priorities and their influence on the uptake and quality of eye care services. This provides an advantage over the ECSAT tool which does not consider patient reported outcomes. The involvement of public input has been considered in previous frameworks, for example the framework of Levesque et al.,^161^ which investigates access to healthcare from the demand and supply side of healthcare. However, this framework is not specific to eye care. Therefore, it does not cover aspects mentioned in our framework, such as the burden of eye disease and the eye care workforce providing the service, as it focuses primarily on the access dimension of healthcare. The framework proposed in our paper combines aspects specific to eye and vision care, as well as provider and service user perspectives. Thus, it contributes to expanding the existing toolkit available to eye care policy makers and planners.

Frameworks specific to eye care have been developed based on the WHO six building blocks advocating for the integration of eye care services into the health system.^162^ However, they have not been tested internationally and require further research and testing in various regions to assess their overall transferability. They^162^, ^163^ also do not consider the detailed interdependency of different domains and how they may influence one another. Our proposed framework holistically captures the inter‐dependency between the different components that influence an eye care service including its impact on the prevalence of eye conditions. The framework has also emerged from the results of our scoping review which are specific to Pakistan. Therefore, for this reason, our framework may provide an advantage over others when evaluating eye care services in Pakistan. The broad nature of our framework gives it potential to be adapted and applied in a variety of ways to evaluate local eye care services. The suggested framework may require adjustments when being applied in different countries, although due to its broad and versatile nature we believe it has the potential to be easily altered to suit a variety of cultural settings.

In addition to addressing a WHO priority area, the review contributes to a number of UN SDGs, which, by definition, have global relevance. It addresses issues within the eye health system, such as inequality within cities and provinces, lack of human resources and varying levels of service quality influenced by socioeconomic status, literacy levels, affordability and accessibility. Furthermore, the economic effect of blindness was discussed, emphasising how blindness and visual impairment negatively affect national economic productivity.^29^

Similar problems with eye health systems have been identified in South Africa, where resources are scarce and inequitably distributed, resulting in varying levels of eye care service quality. There is also a lack of funds dedicated to eye care, and the scope of optometry is limited, for example in terms of binocular vision assessments and contact lens practice.^164^ Inequity in distribution of eye care services has also been witnessed in Latin America where the level of training and distribution of ophthalmologists are highly unequal, with a disproportionate number concentrated in more developed, socially advantaged areas.^165^ Furthermore, barriers to uptake of eye care services identified in Pakistan also exist in other countries. For example, a lack of awareness of eye care services was noted as a barrier in Ghana,^166^ Kenya,^167^ Latin America^168^ and Timor‐Leste.^169^ Another barrier to the uptake of eye care services in Ghana,^166^ South India^170^ and Cambodia^171^ was the cost of services. The existence of similar issues facing eye care services and health systems of lower income countries suggests similar solutions will be required, with appropriate adaptions to account for differences between local healthcare systems.

## CONCLUSIONS

At present, there is a scarcity of high‐quality evidence surrounding pathways, patient priorities and economic aspects of eye care in Pakistan. This study highlights important achievements as well as opportunities for research priority setting regarding eye care service and delivery in Pakistan. Addressing these research priorities may help to improve eye care services further across the country, and in turn decrease the prevalence of avoidable blindness.

## AUTHOR CONTRIBUTIONS

**Manal Malik:** Conceptualization (lead); data curation (lead); formal analysis (lead); investigation (lead); methodology (lead); project administration (lead); resources (equal); validation (equal); visualization (lead); writing – original draft (lead); writing – review and editing (lead). **Niall Strang:** Data curation (equal); investigation (equal); methodology (equal); project administration (equal); resources (equal); supervision (equal); validation (equal); visualization (equal); writing – original draft (supporting); writing – review and editing (equal). **Pauline Campbell:** Supervision (supporting); validation (supporting); visualization (supporting); writing – original draft (supporting). **Sven Jonuscheit:** Conceptualization (equal); data curation (equal); investigation (equal); methodology (equal); project administration (equal); resources (equal); supervision (lead); validation (equal); visualization (equal); writing – original draft (supporting); writing – review and editing (equal).

## CONFLICTS OF INTEREST

The authors report no conflicts of interest and have no proprietary interest in any of the materials mentioned in this article. A version of the abstract has been published online in Ophthalmic and Physiological Optics in the article ‘British Congress of Optometry and Vision Science 2021 Abstracts.’ https://doi.org/10.1111/opo.12900

## Supporting information


Supplementary File 1
