# Supplementary material for: Exploring eye care pathways, patient priorities and economics in Pakistan: A scoping review and expert consultation study with thematic analysis
Source: Ophthalmic Physiol Opt. 2022 Mar 23;42(4):694–716. doi: 10.1111/opo.12977 (PMC9310639; doi:10.1111/opo.12977)
Supplement: Supplementary file 1 [file OPO-42-694-s001.docx]

Supplementary File 1

Example of MEDLINE search string

| # | Searches |
| --- | --- |
|  | "eye care pathway*" |
|  | “primary eye care" |
|  | “secondary eye care" |
|  | "eye health" |
|  | "eye care delivery" |
|  | "eye care service*" |
|  | "district comprehensive eye care" |
|  | "blind*" |
|  | (MH "Eye Diseases") OR (MH "Eye Diseases, Hereditary") OR ""eye disease*" |
|  | (MH "Vision Disorders") OR (MH "Visually Impaired Persons") OR "visual impairment" |
|  | “Vision 2020” |
|  | (MH "Vision, Low") OR (MH "Vision Screening") OR (MH "Vision, Binocular") OR "vision" |
|  | "eye care coverage" |
|  | 1 OR 2 OR 3 OR 4 OR 5 OR 6 OR 7 OR 8 OR 9 OR 10 OR 11 OR 12 OR 13 |
|  | (MH "Patient Preference") OR "Patient priorit*" OR (MH "Patient Participation") |
|  | "patient view*" |
|  | (MH "Public Opinion") OR "patient opinion*" |
|  | "patient experience*" |
|  | "personal experience*" |
|  | "patient concern*" |
|  | "patient attitude*" OR (MH "Attitude to Health") |
|  | “mixed method*” |
|  | interview |
|  | “focus group*” |
|  | (MH "Qualitative Research") OR "“qualitative study”" |
|  | "patient knowledge" |
|  | 15 OR 16 OR 17 OR 18 OR 19 OR 20 OR 21 OR 22 OR 23 OR 24 OR 25 OR 26 |
|  | (MH "Economics") OR (MH "Economics, Hospital") OR (MH "Cost-Benefit Analysis") OR "Economic*" |
|  | "feasibility" |
|  | (MH "Healthcare Financing") OR (MH "Financing, Government") OR (MH "Community-Based Health Insurance") OR "financ*" |
|  | (MH "Financial Management") OR "fund*" |
|  | "subsid*" |
|  | (MH "Cost-Benefit Analysis") OR (MH "Costs and Cost Analysis") OR (MH "Hospital Costs") OR (MH "Cost Savings") OR "cost-effective*" |
|  | (MH "Health Expenditures") OR (MH "Employer Health Costs") OR "cost*" OR (MH "Health Care Costs") OR (MH "Drug Costs") |
|  | Cost-utility |
|  | (MH "Insurance") OR "insurance" OR (MH "Insurance, Health") OR (MH "Insurance, Vision") |
|  | 28 OR 29 OR 30 OR 31 OR 32 OR 33 OR 34 OR 35 OR 36 |
|  | (MH "Pakistan") OR "Pakistan*" |
|  | “Punjab" |
|  | "Gilgit Baltistan” |
|  | "Balochistan" |
|  | "Khyber Pakhtunkhwa" |
|  | "Sindh" |
|  | "Islamabad" |
|  | "Rawalpindi" |
|  | "Lahore" |
|  | "Karachi" |
|  | "Faisalabad" |
|  | "Peshawar" |
|  | "Hyderabad" |
|  | 38 OR 39 OR 40 OR 41 OR 42 OR 43 OR 44 OR 45 OR 46 OR 47 OR 48 OR 49 OR 50 |
|  | “Community eye care worker” |
|  | “eye care worker” |
|  | Optician |
|  | (MH "Optometrists") OR (MH "Optometry") OR "optometr*" |
|  | (MH "Ophthalmology") OR "ophthalmolog*" |
|  | (MH "Tertiary Care Centers") OR "subspecialty" |
|  | (MH "Nurse Specialists") OR (MH "Nurses, Community Health") OR "nurse" |
|  | “Task sharing” |
|  | “Task shifting” |
|  | 52 OR 53 OR 54 OR 55 OR 56 OR 57 OR 58 OR 59 OR 60 |
|  | 14 AND 27 AND 37 AND 51 AND 61 |
|  | 14 AND 51 |
|  | 14 AND 27 AND 51 |
|  | 14 AND 37 AND 51 |
|  | 51 AND 61 |
|  | 37 AND 51 AND 61 |
